# Epidemiological and virological investigation of a Norovirus outbreak in a resort in Puglia, Italy

**DOI:** 10.1186/1471-2334-7-135

**Published:** 2007-11-19

**Authors:** Caterina Rizzo, Ilaria Di Bartolo, Marilina Santantonio, Maria Francesca Coscia, Rosa Monno, Danila De Vito, Franco Maria Ruggeri, Giovanni Rizzo

**Affiliations:** 1 Dep. of Pharmaco-Biology, University of Bari, Italy; 2 National Center of Epidemiology and Health Promotion, Istituto Superiore di Sanità, Roma, Italy; 3 Dep. of Food Safety and Veterinary Public Health, Istituto Superiore di Sanità, Rome, Italy; 4 Dep. of Internal Medicine and Public Health, University of Bari, Italy; 5 Dep. of Odontostomatology and Surgery, University of Bari, Italy; 6 Regional Reference Center for Enteropathogens, Puglia, Italy

## Abstract

**Background:**

This paper describes the third large outbreak of Norovirus (NoV) gastroenteritis reported in the Southern Italy region of Puglia.

**Methods:**

A matched case control study was conducted, on 19 July 2005, for investigating risk factors, using a structured questionnaire on food consumption. A multivariate analysis was conducted to estimate the adjusted Odds Ratios. Laboratory and environmental investigation were also performed.

**Results:**

On the day of the study 41 cases were identified and 41 controls were enrolled. Controls were matched for age and gender. The mean age of the cases was 26 years old, and 58% were female. The clinical pattern of the disease was characterised by the presence of diarrhoea (95%), vomiting (70%), abdominal pain (51%) and fever (32%). Of the 41 cases included in the study, the majority (65%) were residents of Northern Italian regions. No food samples were available for testing. The matched univariate analysis revealed that cases were more likely to have consumed raw mussels, eggs or ice cubes made of tap water than controls. In the multivariate conditional logistic regression analysis, having eaten raw mussels or ice became more strongly associated with illness.

All of the 20 faecal samples collected were tested for NoVs. Eighteen stools (90% of total examined) were positive by RT-PCR, and sequence analysis performed onto 3 samples confirmed the presence of a GGII NoV. No test specific for NoV was performed on water or food samples.

**Conclusion:**

The most likely hypothesis supported by the findings of the epidemiological investigation was that illness was associated with raw mussels and ice, made with tap water. These hypothesis could not be confirmed by specific microbiologic testing for NoV in food or ice. The lack of clear knowledge of NoV as a major causative agent of epidemic outbreaks of gastroenteritis in Italy is due to the absence of timely reporting of the cases to the local public health offices and the uncommon practice of saving clinical samples for virological analysis after bacteriological testing.

## Background

Norovirus (NoV) constitutes one of the four genera of the *Caliciviridae *family [1]. Based on the genetic divergence in the polymerase and capsid genes, NoV are classified into five major genetic groups, called genogroups (GGI through GGV). Most strains affecting humans belong to GGI and GGII, both of which encompass at least 15 genetic clusters or genotypes [2]. GGIV includes a single strain derived from human, GGIII is only represented by bovine strains [2,3], and GGV is a recently reported murine strain being the only NoV able to replicate in cell cultures [4].

NoV is the major cause of non-bacterial gastroenteritis in industrialised countries, and has been shown to account for up to 80% of outbreaks of gastroenteritis in people from all age-groups [5,6]. It is also considered to be the most common cause of epidemic food-borne and waterborne gastroenteritis [7,8]. Transmission occurs person-to-person by faecal-oral route or aerosol formation. Different factors contribute to the high impact of disease caused by NoV, such as a very low infectious dose, the absence of long lasting immunity, the stability of the viruses in the environment, and the ability to be transmitted by a variety of routes [9]. Therefore, outbreaks caused by NoV may involve very large number of people in communities such as restaurants, tourist resorts, cruise ship, hospitals, schools and nursing homes [10-15].

In Italy, according to the statutory system for notification of infectious diseases, all outbreaks are to be reported [16]. Nevertheless, as shown for the case-based reporting on other infectious diseases, underreporting is frequent also for outbreaks, and varies across the country [17]. To date only three reports have described the involvement of NoV in the etiology of GE epidemics in Italy [12,18,19]. Few additional studies have described sporadic cases of GE among children [20-22]. Therefore the burden of NoV infection in Italy is presently unknown, and little information is available about circulating viral strains.

In the present study, we describe the investigation of a large outbreak involving approximately 400 guests in a Resort of Puglia region during a three-week period (2–19 July 2005). This represents the third GE NoV outbreak reported in this area of Southern Italy since 2000.

## Methods

### Outbreak investigation

**Figure 1:**
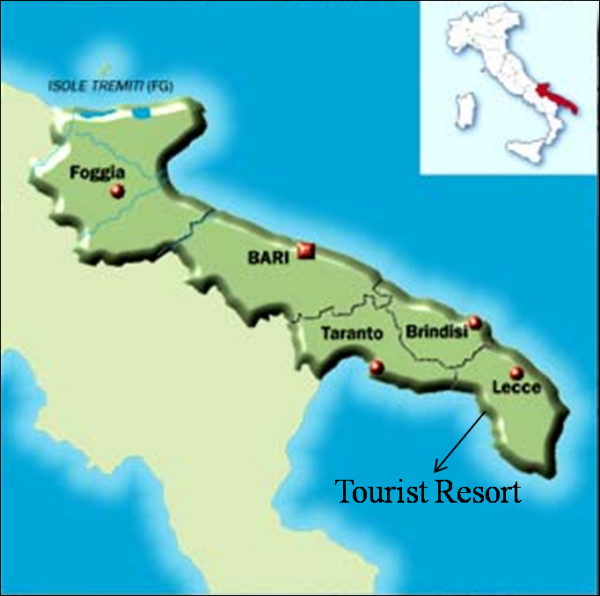
Map of Italy, showing location of tourist resort on Lecce province.

The outbreak occurred at a Hotel in Ugento, a small town in the province of Lecce, Puglia, in Southern Italy during July 2005 (Figure 1). The Hotel area was organized in one single building with 255 guest rooms. The total number of guests during the outbreak period (3 weeks, from 2–19 July) was approximately 400, with arrivals every week on Saturday and departure one or two weeks later. A restaurant and a swimming pool are located in the centre of the building area.

On July 19, 2005, the Regional Centre for Surveillance of Enteric Pathogens in Puglia was alerted by the local health unit that an outbreak of gastroenteritis had occurred at the Hotel. The outbreak continued for about 1 further week. An epidemiological investigation was performed in order to assess risk factors associated with illness.

A case of GE was defined as a guest who had stayed at the resort between 2 to 19 July, and presented two or more loose stools and/or vomiting in 24 hours before interview. A convenience sample of 41 cases and 41 controls were interviewed. Case and control finding was done by a door-to-door search within the Hotel.

A matched case control study was conducted on the 19^th ^of July and trained interviewers collected data using a structured questionnaire. The questionnaire included information on food consumption for investigating risk factors during the last 72 hours before onset of symptoms, and nature, time of onset and duration of symptoms. One matched control was selected for each case (assuming 25% exposure among controls, 80% power to detect a minimum Odds Ratio of 4.1, alpha error of 5%) matched for age (+/-2 years) and sex. Controls were excluded if they reported to have experienced an episode of gastrointestinal illness (two or more loose stools in a 24 hour period, or vomiting, or abdominal pain) within seven days prior to the onset of illness in the matched case. If cases or controls were below 16 years of age, their parents or guardians were interviewed.

### Statistical Analysis

All questionnaires were collected in the Regional Centre for Surveillance of Enteropathogens in Puglia, where data were entered into a File-Maker Pro database (FileMaker, Inc.; Santa Clara, CA, USA). Categorical variables were compared using the chi^2 ^test; continuous variables were compared using the Wilcoxon Mann-Whitney test. In the univariate analysis, exposure to potential risk factors was compared between cases and controls calculating matched "Mantel-Haenszel" Odds Ratios (mOR) and exact 95% confidence intervals (95% CI).

A multivariate conditional logistic regression model was then performed to assess independent effects of the exposure variables and to estimate adjusted odds ratios (aOR); risk factors associated with the outcome (P < 0.20) in the univariate analysis, after testing for multi-colinearity, were considered eligible to be included in the multivariate model, and retained in the final model, together with matching variables, according to a log likelihood-ratio test for goodness-of-fit. For each variable, the model excluded records with missing values. Analysis was carried out using STATA 8.2 (Stata Corp, College Station, Texas, USA).

### Laboratory investigations

Twenty faecal samples were collected from 41 guests whose symptoms matched the case definition. Ova and parasites were investigated by direct microscopy, and Salmonella, Shigella, Campylobacter, *Yersinia enterocolitica*, *Staphylococcus aureus*, and enteropathogenic *E. coli *were sought by standard methods [12]. The presence of *Clostridium perfringens *enterotoxin (CPE) was investigated either by assaying the cytopathic effect on Vero cells or by reverse passive latex agglutination (RPLA) test (Oxoid Italia Spa, Garbagnate Milanese, Milan) according to the manufacturer's instructions [12].

All faecal samples were tested for NoV by ELISA test (Dako Cytomation, Ely, UK) to detect NoV antigen and by RT-PCR. ELISA assays were performed using a 1:10 (w/v) dilution of stools in kit sample buffer following the Manufacturer's instructions.

Viral RNA was extracted from 150 μl of 10% water suspensions of the stool specimens with the Nucleospin RNA Virus extraction kit (Macherey Nagel, Germany), according to manufacturer's instructions. RNA was stored at -80°C until use in RT-PCR assays.

Complementary DNA (cDNA) was prepared from 1.5 μl of eluted nucleic acid by reverse transcription using iScript Reverse Transcriptase and random hexamers (iScript cDNA Synthesis Kit, BioRad Inc., Milan, IT) at 42°C for 45 min. One μl of cDNA was then amplified by PCR, using 20 pmol of primers JV12 and JV13 [23,24] targeting a 327 bases of the RNA-dependent RNA polymerase (RdRp; ORF1) gene of NoV and 1 unit of Taq DNA polymerase (Fermentas GmbH, Rot, DE) in a PCR reaction mix by Invitrogen (Milan, IT). Conditions for the PCR reaction were as follows: denaturation at 94°C for 2 min, 40 amplification cycles including a denaturation step of 1 min at 94°C, primer annealing at 37°C for 1 min 30 s, and extension at 72°C for 1 min, followed by a final extension at 72°C for 7 min. Stools resulting positive by ORF1 RT-PCR were also tested by an RT-PCR targeted onto the ORF2 which codes for the major capsid protein (VP1), using the two primers pairs G2SKF-G2SKR and G1SKF-G1SKR (338 bp fragment) which are specific for genogroups II and I, respectively [25].

Three positive RT-PCR products obtained were purified by silica membrane using the QIAquick Gel Extraction Kit (Qiagen, Milano, IT), and sequenced by the ABI PRISM BigDye Terminator Cycle Sequencing Reaction Kit, version 3.1, (PE Applied Biosystems, Warrington, UK). The sequences obtained were compared with those of the FBVE data bank [26] using DNASIS Max software (Hitachi Software Engineering Company, Alameda, CA, USA).

### Environmental investigation

After July 20^th^, water samples were consecutively collected from various sites, in sterile condition, inside the resort and examined by the Local Agency for Environment. Water samples were subjected to culture tests for enteric bacterial pathogens, according to standard methods; viral contamination was not checked. No food leftover was available for microbiological investigations, at the time of the epidemiological investigation.

## Results

### Outbreak investigation

Four hundred individuals were guest of the hotel, during the entire outbreak period (from 2–19 July). On the day of the epidemiological investigation (July 19^th^) approximately 150 guests were present in the hotel, and 41 (27.3%) cases were found by the door-to-door search.

The epidemiological investigation was performed on 41 cases and 41 controls. The mean age was 26 years old (range 3–58), and 58% of cases were female. The clinical pattern of the disease was characterised by the presence of diarrhoea (95%), vomiting (70%), abdominal pain (51%) and fever (32%). Of the 41 cases included in the study, the majority (65%) were residents of Northern Italian regions.

The matched univariate analysis revealed that the cases were more likely to have eaten eggs, raw mussels or ice cubes made of tap water than the controls. They were less likely to have eaten ham, grilled sausages, snacks, or grilled meat (Table 1). In the multivariate conditional logistic regression analysis, having eaten raw mussels or ice, made by tap water, became more strongly associated with illness (OR = 16.4; 95% CI 1.8–250.9 and OR = 25.5; 95% CI 1.5–442.9 respectively).

**Table 1:** Matched univariate analysis (matched "Mantel-Haenszel" odds ratio: mOR) and multivariate conditional logistic regression analysis (adjusted odds ratio: aOR). Cases of Norovirus infection (n = 41) and Controls (n = 41) according to investigated risk factors

			Univariate analysis			Multivariate analysis		
	
Risk factors(*)	Cases (%)	Controls (%)	mOR	95% CI	*p*-value	aOR	95% CI	*p*-value
Ice	21/41 (51)	12/41 (29)	4.1	(0.9–7.1)	0.04	16.4	(1.8–250.9)	0.04
Eggs	2/41 (5)	8/41 (19)	2.3	(0.1–1.7)	0.12	-	-	-
Grilled sausage	21/41 (51)	25/41 (61)	0.7	(0.2–1.7)	0.17	-	-	-
Ham	1/41 (2)	5/41 (12)	2.8	(0.1–1.7)	0.09	-	-	-
Grilled meat	11/41 (27)	15/41 (37)	3.5	(0.1–1.1)	0.06	-	-	-
Snacks	20/41 (49)	19/41 (46)	0.1	(0.4–2.8)	0.15	-	-	-
Raw mussels	22/41 (54)	13/41 (31)	3.9	(0.9–6.8)	0.04	25.5	(1.5 – 442.9)	0.03

(*) In the univariate analysis, only Risk Factors with p-value<0.20 are reported; in the multivariate analysis, only variables selected by the conditional logistic model according to a log-likelihood-ratio test for goodness-of-fit are reported.

### Laboratory investigations

Two stool samples obtained from 20 patients, resulted positive for GII antigens using the NoV-specific ELISA test. The 20 stool samples were also examined by NoV-specific RT-PCR. Eighteen (90%) samples, including the two positive with the ELISA test, had an amplified DNA of the expected size for both the viral RNA-dependent RNA polymerase and the major capsid protein. All 18 samples resulted positive by genogroups II-specific ORF2 RT-PCR, whereas none was positive for genogroup I.

Three of the amplified DNAs were subjected to sequencing of the diagnostic fragment in the ORF1 polymerase gene and/or in the ORF2 capsid gene. Sequences obtained were aligned with those present in the FBVE database and in NCBI. Analysis revealed that all sequences had a best fit with a GII Norovirus recombinant strain sharing ORF2 GII.3 and ORF1 GII.b characteristics. All samples from the outbreak were identical suggesting the involvement of a single virus strain.

### Environmental investigation

Water samples collected on July 20 from faucets in the bar, the kitchen, and a guest room had high levels of coliforms (up to 130 CFU/mL) and fecal streptococci (up to 22 CFU/mL). After microbiological analysis for routine bacterial enteropathogens, samples were discarded impairing possible virological investigation.

## Discussion

This is the third outbreak described in a tourist resort in Southern Italy [12,18] to date. The outbreak occurred during three weeks in July 2005. The delay in reporting the epidemics to the local Public Health Unit only allowed to investigate cases during the last week. Moreover, data concerning the beginning of the outbreak are limited to those reported from the Local Health Unit (personal communication). No further cases were reported after July 29, when specific public-health measures were implemented (such as drinking only bottled water, super-chlorination of the resort tank, stopping the serving of raw mussels, and disposal of previously stored ice).

Infection appears to have spread rapidly within the resort, hitting persons within just a few days after their arrival, similarly to our previous observations during the 2000 epidemics in Italy [12].

During the period of the outbreak, no other NoV outbreaks were notified outside the resort in the same area. Moreover, a recent study in a close area (Taranto province, see Figure 1) has shown that during 2005 the number of patients with gastroenteritis seeking hospital care was lower compared to 2006 [27].

The most likely hypothesis supported by the findings of this epidemiological investigation was that illness was associated with raw mussels and ice made by tap water. However just over half of the cases reported eating raw mussels, eggs and/or ice. There are a number of possible explanations for cases not reporting to have eaten the implicated items. Interviews took place during the outbreak period, but the food buffet served to residents changed weekly during the three weeks of the outbreak and answers given were indeed food preferences rather than food actually eaten. Several cases who did not report the consumption of ice were children aged between 2–8, and parents or guardians who answered on behalf of their children may not have been aware of all food items eaten during a holiday period with several annexed social gatherings. Besides, cases might have been secondary cases, resulting from person-to-person transmission.

Raw mussels and ice are a plausible vehicle for infection as described before in Italy [12,18]. Nonetheless, it should be noted that no food leftover was available for virological investigations. Also in the case of water and ice samples the presence of virus could not be confirmed by specific laboratory analysis. Although laboratory testing for NoV could not be performed on drinking water, the presence of faecal bacteria suggests that the water system may have been the actual source of NoV. As we were able to investigate only the final week of the outbreak, it cannot be ruled out that the association between water and cases may indeed reflect contamination of water with patients stools from within the hotel late during the epidemic. Such a hypothesis appears however unlikely since the environmental inspection did not identify any failure in the water system of the resort. Rather, the observation that tap water samples from different places in the resort showed contamination with faecal bacteria is suggestive that the water pipeline was polluted ahead of the resort. It cannot be ruled out that a possible food item contaminated with NoV may have been involved in some phase during the outbreak, and may have spread the virus to other foodstuff or environment. In particular, it is possible that mussels may have been rinsed with clear water before consumption raw or lightly cooked, although this could not be decisively ascertained from interviews to cooking personnel. In addition to a point source of infection, a person to person transmission of Nov during the outbreak is also likely to have occurred as supported in particular by both the occurrence of few isolated cases in the first days of the outbreak before the peak four days later, and the occurrence of secondary cases within families. Multiple transmission routes have been reported previously [12].

A conclusive association of the outbreak with noroviruses is supported by the results of laboratory investigations. We used both NoV-specific ELISA and RT-PCR to test 20 faecal samples collected during the outbreak investigation. Using ELISA test, 2 (5%) of the 20 samples were positive for GII antigens. The RT-PCR test detected NoV RNA in 18 (90%) samples, including the two positive by ELISA, out of the 20 cases. These results fit with previous findings that ELISA assays have a lower sensitivity as RT-PCR methods [28], although ELISA might represent a simple and rapid diagnostic test for timely investigations of NoV outbreaks in laboratories with low capacities. Sequence analysis of the amplified fragments of the ORF1 and ORF2 from 3 samples showed that a recombinant GIIb(ORF1)-GII.3(ORF2) NoV strain [29,30] was the causative agent of the outbreak. That is not surprising since most GII.b NoV strains described in the literature appear to be recombinant NoV genotype [31,32]. Sequences from different patients shared 98–100% nucleotide identity, that supports the involvement of a single strain at least during the third week of the outbreak. The spread of GIIb strain of NoV has been previously reported in Italy in 2002, in association with sporadic cases of gastroenteritis in children requiring hospitalization [21]. GIIb strains have circulated in several European countries since 2000 [29], and have been associated with outbreaks in schools, nursing homes, rural villages and water-borne outbreaks [14].

## Conclusion

The delay in reporting the outbreak is the main cause why it was not possible to identify the source of infection, since most of the patients during the first period of the epidemic were no longer available for interviewing. The data collected by interviews suggest that NoV had spread through consumption of ice made by tap water and raw mussels. Before July 23, no control measures to limit the spread of the infection were implemented, probably because the local health unit did not address the point source and failed to prevent person-to-person transmission. After July 23, the consumption of tap water was banned, because of the faecal contamination, and only bottled mineral water was served in the resort restaurant and used to wash vegetables. Water from the main tank, however, continued to be used for showers. The water inside the resort tank underwent superchlorination. However, NoV does survive high levels of chlorination [12], and the treatment was performed only once, at a late stage of the outbreak.

In Italy, obtaining knowledge about the relative role of NoV as causative agent of outbreaks of gastroenteritis is hampered by the huge underreporting as well as late reporting of cases, and by disregard to collect and save clinical and environmental samples for virological testing. Moreover, this outbreak clearly shows the lack of laboratory capacity locally to test water and food for NoV, that is presently available only at the National level [12]. The surveillance and outbreak management of NoV as a major entero-pathogen involved in gastroenteritis should be improved, by stimulating diagnostic testing for viruses and timely reporting of cases to the local health authorities.

## Competing interests

The author(s) declare that they have no competing interests.

## Authors' contributions

CR designed the epidemiological study, performed the statistical analysis and drafted and edited the manuscript. MS, FMC, RM performed the laboratory test on human samples. MS, IDB carried out the molecular studies, participated in the sequence alignment and drafted the manuscript. DDV, FMR and GR conceived the study, and participated in its design and coordination and helped to draft the manuscript. All the authors read and approved the final version of the manuscript.

## Pre-publication history

The pre-publication history for this paper can be accessed here:


